# Pandemic-Related Stress Symptoms Among Norwegian Parents of Adolescents in Grades 6 to 8

**DOI:** 10.3389/fpsyt.2021.701782

**Published:** 2021-10-06

**Authors:** Thormod Idsoe, Atle Dyregrov, Harald Janson, Ane Nærde

**Affiliations:** ^1^The Norwegian Center for Child Behavioral Development, Oslo, Norway; ^2^Center for Crisis Psychology, University of Bergen, Bergen, Norway

**Keywords:** COVID-19 lockdown, pandemic-related stress-symptoms, PTSD symptoms, parents, IES

## Abstract

We investigated pandemic-related stress symptoms during the first COVID-19 lockdown period in spring 2020 among parents of adolescents that were 11 to 13 years old in the study period. We also investigated whether parental stress symptoms were associated with family situation and family activities during lockdown. Altogether 147 couples reported about their own trauma-related stress symptoms following the outbreak of the pandemic. Among the respondents, 9.5% of the mothers and 10.2% of the fathers had scores over cutoff on the screener (IES-6) measuring stress symptoms, a non-significant gender difference. Scores on the screener were not associated with family contamination or lockdown consequences. Family activities during lockdown did not impact the pandemic stress symptom levels. Whereas, the experience of the COVID-19 pandemic pose a stressor to most people, it is unlikely to be a criterion A event for other than directly affected families.

## Introduction

The ongoing COVID-19 pandemic may lead to stress symptoms in the same way as previous viral outbreaks (1). It is well-known that stress symptoms can have serious consequences for mental health and normal functioning. The occurrence of such symptoms related to the COVID-19 outbreak should therefore be investigated to identify potential needs for intervention. As family conflict typically peaks when youth enter early adolescence (2), parents of youth may be particularly vulnerable to pandemic stress. Our aim was thus to investigate pandemic related stress in this group of parents.

The World Health Organization (WHO) officially declared the COVID-19 as a pandemic on March 11, 2020 (3). This triggered a series of national actions taken in many countries to prevent the spread of the virus, including social and physical distancing, hygiene measures (handwash, face masks etc.), closure of day-care centers, schools and universities, and encouragement to work from home when possible (4, 5). Due to the rapid spread of the virus and the lack of evidence-based knowledge about transmission and infection control, the Norwegian society was in partial or full lockdown from March 12 to the end of April 2020. From March 12, day-care centers, schools and universities were closed, replaced by digital solutions and home schooling, and people were obliged to work from home whenever possible. Cultural and sports events, as well as organized sport activities both indoors and outdoors, were prohibited. Strong restrictions were introduced regarding social meetings and private visits. Although no curfews, outdoor people had to keep at least one meter distance to others, and gatherings were restricted. Indoor, people should keep at least two meter's distance (except within family) and visits should be avoided (e.g., grandparents were not allowed to meet with their grandchildren). By the end of April, day-care centers and schools gradually reopened, although with somewhat varying local restrictions and rules for attendance. This means that the first national lockdown lasted for about 7 weeks. We gathered our data between April 8 until July 7, although most of the participants answered in the first part of this period.

Parent-adolescent conflict peaks in frequency in early adolescence (2, 6), possibly related to more autonomy-seeking behaviors among adolescents in this period (7). However, high conflict levels in early adolescence may also be related to adolescents experiencing more intense emotions in this phase of life as concluded in a recent review by Bailen et al. (8). As a consequence, many parents may face more difficulties in their relations with their youngsters during this period. The mentioned restrictions could potentially challenge parent-child relations during the pandemic. Potential parental PTSD symptoms due to the threat of the pandemic could affect developmental outcomes in adolescents. Although this could be a direct effect (by adolescents observing, learning and internalizing symptoms), Samuelson et al. (9) found that the effect was mediated by parenting stress (e.g., poorer parenting and supervision). This is in accordance with the Family Stress model (10) that explains how stressors can lower the quality of parenting that again disturbs developmental outcomes for the adolescents. Positive parenting may also be a protective factor (11), and family activities like meals have been found to be a protective factor for substance use among adolescents (12). On this background, we were interested in investigating pandemic-related stress symptoms among this group of parents.

Meta-analyses have shown that previous viral outbreaks have been associated with several kinds of mental health problems, among them posttraumatic stress disorder (PTSD) (1, 13). PTSD is different from most other psychiatric disorders insofar as there is an established link between exposure to (a) traumatic event(s) and resulting symptoms (14). Eight diagnostic criteria are listed in the DSM-5 (labeled A through H). The A criterion (stressor) states that the person was exposed to “actual or threatened death, serious injury, or sexual violence” (p. 271). The exposure can be direct, as a witness, by learning that a relative or close friend was exposed, or indirectly by being exposed to aversive details of the trauma (e.g., as first responders medics). Symptoms emerge within four clusters, “…intrusion symptoms associated with the traumatic event(s)…” (p. 271) (B—intrusion symptoms), there is “Persistent avoidance of stimuli associated with the traumatic event(s)….” (p. 271), such as thoughts, feelings, external reminders (C—avoidance), there are negative thoughts or feelings that began or worsened after the trauma [D—“negative alterations in cognitions and mood….” (p. 271)], and there is trauma-related arousal and reactivity that began or worsened after the trauma [E—“.alterations in arousal and reactivity.” (p.272)]. Additionally, it is required that symptoms last for “…more than 1 month…” (p. 272) (F—duration), that symptoms “…causes clinically significant distress or impairment in social, occupational, or other important areas of functioning” (p. 272) (G—functional significance), and that symptoms are not “attributable to the psychological effects of a substance (e.g., medication, alcohol) or another medical condition” (p. 272) (H—exclusion). Self-report screening questionnaires cannot be used for making diagnostic distinctions (for specific diagnostic criteria). However, screening questionnaires may indicate symptom cluster severity levels for intrusion, avoidance and arousal like in “The impact of event scale” (15). For our study the A criterion (stressor) is of special relevance. Is it possible that parents of early adolescents can perceive the COVID-19 pandemic as a Criterion A event? According to the DSM-5 (14), Criterion A refers to exposure to “actual or threatened death, serious injury, or sexual violence,” and it has been questioned whether the COVID-19 pandemic satisfies this criterion (16–19). Van Overmeire (19) is critical of considering COVID-19 as a traumatic event by itself, arguing that peoples' experiences with COVID-19 vary greatly, from non-threatening experiences to actual infection and fear of death. However, this variation was investigated by Bridgland et al. (16) who asked participants in five western countries (*N* = 1,040) to indicate COVID-19 events they had been directly exposed to, events they anticipated would happen in the future, and other forms of indirect exposure such as through media coverage. Following this, they measured PTSD symptoms in relation to COVID-19. They reported 13.2% of their sample to be likely PTSD-positive, despite types of COVID-19 “exposure” (e.g., lockdown) not fitting DSM-5 criteria. Based on the uncertainty as to whether COVID-19 satisfies criterion A, we chose to label the symptoms we assess as “pandemic-related” instead of “posttraumatic.”

Some studies, [e.g., (20–22)] have reported COVID-19-related posttraumatic stress symptoms from different populations (students, hospital staff, but also from the general population). As we study the general population, we will briefly review previous relevant investigations, keeping in mind that comparison across countries with different rates of COVID-19-related illness and death, different methodology, and different actions taken to intervene, is challenging. One specific issue is that some of the studies have re-formulated the items supposed to assess PTSD symptoms by referring directly to COVID-19. For example, a question from “The Impact of Event Scale—Revised” (IES-R) assessing avoidance is re-formulated from “I tried not to think about it” to “I tried not to think about the corona situation.” This is an attempt to refine the A criterion (stressor) (14), i.e., to reduce the simultaneous impact of other potentially traumatic experiences that could also cause PTSD symptoms. As this may affect the reported symptom levels, we have described how the studies reviewed below handled this important issue. It is also possible that the severity of the particular national lockdowns will affect the parent-child relationship, by influencing levels of PTSD symptoms. This should be kept in mind when comparing results across countries.

From Italy we review three relevant studies. The Italian Government implemented extraordinary measures to minimize contact with people infected by COVID-19 and thereby limit viral transmission from March 8th, 2020. These measures included social isolation, restrictions on movements and in some cases also formal mandatory quarantine. Many were detained at home, only allowed to leave their houses if strictly necessary, and they were only allowed to go to work if physical presence was strictly demanded. Surgical masks were mandated in public places. Usual Italian lifestyle and social relationships were completely changed (23). The situation in Italy was dramatic. It quickly became one of the countries with highest levels of COVID-19 infections. About 140.000 had been infected by COVID-19, and ~17,000 had died by April 8th 2020 (23). Casagrande et al. (23) investigated symptoms of posttraumatic stress disorder (PTSD) among 2,291 respondents (74.6% females) in the Italian population during the lockdown period from March 18th to April 2nd 2020. Age range; 68.6% between 18 and 29 years old, 21.2% between 30 and 49 years old, and 10.3% from 50 years old. The study was a cross-sectional survey using different platforms and social media to gather data. A COVID-19 modified version of PTSD Checklist for DSM-5 (24) was used to assess specific symptoms concerning the COVID-19 emergency, similar to PTSD symptoms, according to DSM-5 criteria. Analyses revealed that 7.6% of all respondents reported high levels of COVID-related PTSD, with a score higher than 1.5 standard deviations from the mean score. In general, some factors (e.g., younger age, being female, having uncertainty about the possibility of contracting the infection by COVID-19, or having had direct contacts with people infected by COVID-19) were associated with higher levels of mental health problems. In another study of the Italian population, Rossi et al. (25) gathered information from a large sample (*N* = 18,147, 79.6% females, median age = 38) in the lockdown period between March 27th and April 6th. They found that as many as 37% of the sample reported posttraumatic stress symptoms that were regarded as clinically relevant. These researchers used logistic regressions to identify several factors that had an impact on symptom levels, including being a woman [odds ratio (OR) = 2.12, i.e., being a woman more than doubled the chance of having a one-unit higher symptom score], younger age (OR = 1.49), coming from Southern Italy (OR = 1.36), being under quarantine because infected (OR = 1.74), having experienced a stressful life event due to COVID-19 (OR = 1.46), discontinued working activity (OR = 1.15), having a loved one being infected (OR = 1.68), and having a loved one deceased (OR = 1.22). These findings illustrate the importance of going beyond prevalences only and investigate other variables which may impact the symptom levels. For assessment they used the posttraumatic stress symptoms subscale (GPS-PTSS) of a newly developed measure—the Global Psychotrauma Screen (26). Although initial data on this brief instrument has provided a first indication that the measure is valid (26), cross-cultural validity has not been established. This makes it hard to compare their results with other findings. Furthermore, it does not seem like the items used to measure PTSS are specifically COVID-19 formulated. In a third study from Italy conducted from March 19 to April 5, 2020 (in the Italian lockdown period), Castelli et al. (27) investigated the general Italian population. The sample had a mean age of 35.1 (SD 14) years, 69% were females, and 71% came from Northern Italy. The findings revealed that 20% (more females than men) reported significant PTSD symptoms (over cutoff on PTSD Checklist for DSM-5, PCL-5). These authors also found several factors that predicted the likelihood of PTSD symptoms like gender (higher for females), education level, contact with individual(s) positive for COVID-19, life satisfaction, and health concern. The authors conclude that this indicates that the respondents likely experience the COVID-19 outbreak as a psychological trauma with possible effects of both infection fear and isolation measures taken by the government to contain it. The authors point to the danger that PTSD symptoms may develop into PTSD for some of those affected, and they recommend screening to identify people at risk. There is no information as to whether the items or the introductory text were COVID-related.

We have also reviewed three relevant studies from China. First, Liu et al. (28) investigated prevalence of posttraumatic stress symptoms (PTSS) 1 month after the December 2019 COVID-19 outbreak among residents in Wuhan and surrounding cities. These were the hardest hit areas, and according to the authors, the residents faced high risks of infection. The questionnaires were sent to 285 participants (54.4% females), 47.7% between 18 and 35 years old, while 52.3% were >35 years old. In addition, the authors claim that information about the virus from the media was not clear and definite as the number of infections were increasing, and that this created an insecurity among Chinese people, especially in the Wuhan area. This insecurity was reinforced by shortage of medical personnel and lack of resources including masks and protection equipment. So, these inhabitants most likely perceived the situation as quite dramatic. Using the PTSD Checklist for DSM-5, the research group identified 7% that met the criteria for PTSD. Gender and educational levels were significantly linked with symptom levels, as well as what was called “population susceptibility,” meaning whether participants were classified as from the general public, having had close contact with contaminated, being health care worker, or being a confirmed or suspected case of infection. From their report there is no indication that the items used to measure PTSS were specifically COVID-19 formulated. In another study from China, Wang et al. (29) investigated immediate psychological responses during the initial stage of the COVID-19 period (from January 31st to February 2nd 2020) in the general population. This investigation took place about the same time as the one by Liu et al. (28). Based on a sample of 1,210 participants (67.3% women) aged 21.4 to 30.8 years from 194 cities in China, as many as 53.8% of respondents rated moderate or severe psychological responses (score > 33, indicating clinical range symptoms) impact of the situation according to the IES-R (female gender was significantly associated with higher scores). Even though the measurement instrument (IES-R) is reported to be well-validated for the assessment of psychological impact after exposure to a public health crisis, it is not clear whether the items or the introductory text in this study were COVID-formulated. The difference in the prevalence between the studies of Liu et al. (28) and Wang et al. (29) is difficult to explain, but could possibly be related to different measures as well as different sampling procedures. Symptom levels were higher for females, students, and individuals with poor self-rated health status. In a third Chinese study, Zhang and Ma (30) conducted a cross-sectional study of the impact of the pandemic among 263 (59.7% females) local residents in Liaoning Province, China, from January 28 until February 5 2020. The mean age of the participants was 37.7 ± 14.0, and 41.4% were between 18 and 30 years. Using the original 15-item version of the IES, they found that 7.6% of the participants had a score indicating high symptom levels, scores similar as in the study by Liu et al. (28). None of the sociodemographic variables were associated with the IES score. Unfortunately, it is not clear whether the items or the introductory text of the IES used in this study were COVID-formulated.

In a recent study from Norway, Bonsaksen et al. (31) investigated PTSD symptoms among 4527 respondents (85% women) aged 18 or higher in the general population between 8 April 2020 and 20 May 2020 by use of the PTSD Checklist for the DSM-5, linking items specifically to the COVID-19 pandemic. Prevalence of symptom-defined PTSD was 12.5% for men and 19.5% for women (this difference was statistically significant). Furthermore, high prevalence was associated with lower age, lack of social support, and a range of pandemic-related variables such as economic concerns, expecting economic loss, having been in quarantine or isolation, being at high risk for complications from COVID-19 infection, and having concern for family and close friends. They concluded that posttraumatic stress reactions appeared to be common in the Norwegian population in the early stages of the COVID-19 outbreak. Our study will add to their findings by investigating parents of children in early adolescence.

In conclusion, the COVID-19 pandemic is associated with stressful impact in all studies reviewed. However, the magnitude varies across studies, probably due to the populations under study and the assessment instruments utilized. Most populations studied, e.g., the Italian, experienced more severe outbreaks than the Norwegian, with more deaths and severe cases of illness. Often it is unclear whether the wording and instructions used is COVID-formulated.

Our research questions for the present study were: (1) What are the levels of pandemic-related stress symptoms among parents of adolescents in grades 6 to 8 during the first lockdown period in spring 2020 in Norway? (2) Is family situation (e.g., home schooling, contamination, etc.) and family activities during lockdown related to levels of stress symptoms?

## Materials and Methods

### Sample and Procedure

The Behavior Outlook Norwegian Developmental Study (BONDS) is a longitudinal study that follows 1,159 children's (51.8% boys) social development from they were 6 months onwards—for a more detailed description, see Nærde et al. (32). The families were recruited in 2006–2008 through public child health clinics in five Norwegian municipalities. Parents of a total of 1,159 children (60% of 1,931 eligible) gave their consent to participate, and the retention rate in the study has been quite high throughout the first years, with 98% of families participating at 1 year, 93% when the children were 4, and 82% in first grade (when the children were ~6 years old). The children were aged 11–13 years in 2020 when the data for the current study were collected. Altogether 616 parents, who already participated in the regular data collection in spring 2020, were invited to participate in this extra investigation about Coivd-19. We received answers from 312 mothers and 201 fathers. Of those, 147 were couples, and this was our study sample. Our sub-sample consisted of parents of more girls (53.1%) compared with the remaining part of the larger sample (47.5%). We also compared A SES index for early life socioeconomic risk (based on education, employment, financial hardship, and housing), and our sub-sample had a significantly higher score on this index. Consent was obtained electronically, and all parents provided informed written consent. This investigation has been approved by The Norwegian Regional Committee for Medical and Health Research Ethics (South East), approval reference: 12552 “Barns sosiale utvikling – fortsettelse.”

### Instruments

#### PTSD Symptoms

PTSD symptoms were measured by use of a modified version of *the Impact of Events scale* −*6* (IES-6) (33), in which the instruction as well as four of the six items specifically framed the COVID-19 pandemic as the target event (Tables 1, 2). The IES-6 is an abbreviated version of the 22-item scale IES-R (15). The respondents reported how bothered or stressed they were (over the past 7 days) by symptoms related to the COVID-19 pandemic, rating themselves on a Likert scale: “not at all” (item score 0), “a little bit” (score, 1), “moderately” (score, 2), “quite a bit” (score, 3), or “extremely” (score, 4). Principal component analyses with Varimax rotation revealed one-factor solutions (eigenvalue > 1) with standardized factor loadings ranging from 0.50 to 0.88 for mothers and fathers, respectively. The score on the IES-6 is calculated as the average of the six items. Cronbach's alphas were 0.77 for mothers and 0.80 for fathers on the total scale score. Thoresen et al. (33) provided sensitivity and specificity, positive and negative predictive value, and overall efficiency of the unmodified Norwegian version of the IES-6, demonstrating that a cutoff level of 1.75 will indicate a likely diagnosis of PTSD. Even though cutoff scores may vary between different populations, Hosey et al. (34) recommended a cutoff score of 1.75 (average value) for the IES-6 when investigating PTSD among survivors of acute respiratory distress syndrome (ARDS). This is close to the level recommended by Thoresen et al. (33) if the sum score is converted. We used this cutoff (1.75) as a reference for our study.

**Table 1A:** About the situation in your family (mothers' answers).

	**Yes**	**No**			
Have you stayed home most of the time during the last couple of weeks?	113	34			
	Yes	No	Not relevant		
Has your partner/spouse/cohabitant stayed at home most of the time during the last couple of weeks?	96	39	12		
	Yes	No			
Has your work situation changed as a result of the corona situation?	80	67			
	Yes	No	Not relevant		
Has your partner's work situation changed as a result of the corona situation?	61	72	12		
	0 children	1 child	2 children		
How many children of kindergarten age have been home most of the time during the last couple of weeks?	132	13	1		
	0 children	1 child	2 children	3 children	4 children
How many school-age children have been home most of the time during the last couple of weeks?	5	21	90	25	6
	No	Do not know	Yes, presumed infected but not tested	Yes, confirmed infection through test	
Has anyone in the family been infected with corona?	125	18	4		
	No	Yes, some loss of income	Yes, significant loss of income	Do not know	Not relevant
Will you lose income as a result of the corona situation?	121	17	4	5	
Will your partner lose income as a result of the corona situation?	106	20	4	5	12

#### Family Situation

Nine questions were formulated to map the family situation during lockdown. The topics included whether they and/or their partners had to stay at home during the past 2 weeks, whether their work situation and/or that of their partner had changed and how (home office/loosing job/changing job tasks), number of children in kindergarten and school age that were staying at home, whether family members had been contaminated, and finally if they and/or their partner expected to lose income due to the pandemic situation. Answering categories are shown in Tables 1A,B.

**Table 1B:** About the situation in your family (fathers' answers).

	**Yes**	**No**			
Have you stayed home most of the time during the last couple of weeks?	104	43			
	Yes	No	Not relevant		
Has your partner/spouse/cohabitant stayed at home most of the time during the last couple of weeks?	100	35	11		
	Yes	No			
Has your work situation changed as a result of the corona situation?	61	86			
	Yes	No	Not relevant		
Has your partner's work situation changed as a result of the corona situation?	60	71	16		
	0 children	1 child	2 children	3 children	
How many children of kindergarten age have been home most of the time during the last couple of weeks? (Quantity)	127	13	1	1	
	0 children	1 child	2 children	3 children	4 children
How many school-age children have been home most of the time during the last couple of Weeks? (quantity)	9	21	89	23	5
	No	Do not know	Yes, presumed infected but not tested	Yes, confirmed infection through test	
Has anyone in the family been infected with corona?	118	26	3		
	No	Yes, some loss of income	Yes, significant loss of income	Do not know	Not relevant
Will you lose income as a result of the corona situation?	108	21	8	10	
Will your partner lose income as a result of the corona situation?	106	20	4	5	12

#### Family Activities

We constructed an index based on yes/no (yes = 1, no = 0) answers to 20 activities/routines that the family could partake in during the pandemic (e.g., fixed hours for school/play, watching TV together, listening to music together, go for walks, doing physical activities). Maximum possible score = 20, higher scores indicate more activities (Table 2).

**Table 2:** What do you do in the family to cope with the corona situation? Which of the following activities do you (or have you done) in the ongoing the corona situation.

	**Mothers**		**Fathers**	
	**Yes**	**No**	**Yes**	**No**
Have fixed times for activities such as school, play, rest	103	44	84	63
Have times or places to have your own time	52	95	45	102
Have PC-free or TV-free times	32	115	21	126
Playing computer games together	28	119	30	117
Plays board games, card games or similar together	86	61	81	66
Puzzle	49	98	34	113
Watching TV or movies together	132	15	118	29
Listening to music together	32	115	22	125
Reads aloud	27	120	17	130
Cooking together	97	50	88	59
Draws or paints	35	112	25	122
Do needlework	24	123	16	131
Builds e.g., Lego or models	18	129	19	128
Going out/going for a walk together	132	15	122	25
Do physical activities outdoors (running, cycling, exercise, trampoline or other)	104	43	90	57
Do physical activities inside	39	108	39	108
Often talks on the phone with relatives/friends	49	98	30	117
Have video calls on mobile or online with relatives/friends	53	94	47	100
Is a lot on social media	37	110	28	119
Children use social media a lot	46	101	42	105

### Analyses

Data were analyzed by means of SPSS (35). Scale-scores for PTSD symptoms for mothers and fathers were compared with paired samples *t*-test. Because of the ordinal data we used Spearman correlations to investigate associations between PTSD symptoms on the one hand and family situation (contamination or lockdown consequences) and family activities on the other.

## Results

We received answers from both parents in 147 couples (*n* = 294). Average scores for PTSD Symptoms were 0.91 (SD = 0.58) for mothers and 0.82 (SD = 0.68) for fathers, however, this difference was not significant (*p* = 0.229). Furthermore, 9.5% (*n* = 14) of the mothers and 10.2% (*n* = 15) of the fathers scored above the conventional cutoff value (1.75). Descriptive information about family situation and family activities are shown in Tables 1A,B, 2. As evident from the tables, the majority had to work from home during the lockdown period while taking care of their children at the same time. However, few reported contamination by the pandemic virus. More than 2/3 of the sample did expect to lose income because of the pandemic.

We did not find any significant associations between the index for family activities and symptom scores, correlations = 0.136 (*p* = 0.147) and 0.116 (*p* = 0.168) for mothers and fathers, respectively, or items measuring family situation (contamination or lockdown consequences) and symptom scores (non-significant correlations ranging from 0.023 to 0.160).

## Discussion

We investigated pandemic-related stress symptoms in the initial COVID-19 lockdown period during spring of 2020 among parents of adolescents aged 11–13 years old. Among the respondents, 9.5% of the mothers and 10.2% of the fathers had scores over cutoff on the self-report screening questionnaire for stress symptoms. Three previous studies reported similar clinical range scores at about 7 to 8 percent of the respondents (23, 28, 30), while other studies have reported higher percentages ranging from 12.5% (31), 20% (27, 31), 37% (25) up to 53.8% (29). Especially the discrepancy between our study and Bonsaksen et al. (31) is interesting as both are Norwegian studies. However, the discrepancies could be due to different samples. While our study consists of parents of youth in early adolescence—the Bonsaksen et al. study comprised participants aged between 18 and 70+. The fact that this study included older persons may have contributed to the higher PTSD scores, as older persons have higher risk for more severe COVID-19 related symptoms, and thereby could be more distressed and worried by the situation.

Even though the majority of the parents had to work from home and also take care of their homeschooled children during this period, not many had been contaminated with the virus. Also, more than 2/3 of the sample did not expect to lose income due to the pandemic. Based on this it could well be that the parents in our sample did not experience the pandemic as hard as people in China and Italy as reported initially. We believe it is important not to necessarily attribute high pandemic-related stress scores to potential PTSD. This is in accordance with the discussion we refer to initially, reflecting different arguments for considering COVID-19 as a traumatic event or not, and thereby whether it satisfies criterion A for PTSD (14). However, this also touches on the general controversy around the definition and interpretation of criterion A (36–41). Some researchers have suggested that criterion A is not necessary for diagnosing PTSD (37, 38), and studies have indeed found persons exposed to traumatic events reporting similar (42) or even lower levels of the clusters of PTSD symptoms than persons that have experienced “non-traumatic” stressful life events (43). Keeping this in mind, we believe that COVID-19 is not necessarily a traumatic event by itself.

The subjective reactions to the pandemic are likely to fluctuate over time, they ebb and rise with decreasing and increasing national mortality -and infection rates. They furthermore vary according to the prospects of vaccination and the measures put in place by the national and local authorities to reduce contagion. Even though the majority of our data were collected in the initial lockdown period, some of the participants responded from May to July, a period coinciding with the reopening of the Norwegian society. Moving out of lockdown may have brought hope for the future with more optimism and alleviation of stress, anxiety, and worries. We argue for caution in attributing high symptom scores to potential PTSD as a mere result of the pandemic. Even though studies use validated instruments for symptoms of PTSD, the reported symptoms could merely indicate natural worries and reactions to continuous stress, rather than PTSD. Whereas, the experience of the COVID-19 pandemic will pose a stressor to most people, it is unlikely to be a criterion A event for many (44). With the fluctuating nature of the stressor, it might not be traumatic unless a person or family member's life is directly in danger. However, it may represent an oscillating, continuous stressor for the population at large.

Although the Impact of Event Scale was originally constructed to measure posttraumatic stress symptoms, responses to the six items included in the short form are understood in the context of the pandemic and the restrictions imposed. The pandemic has dominated the media and captured the public's attention. Answering affirmatively to the questions posed, e.g., confirming that other things kept making them think about the corona situation, and that they thought about the corona situation when they didn't mean to, may likely be influenced by the constant bombardment of information about the pandemic. It is natural to react with anxiety and worry under such circumstances, and such reactions may contribute to the compliance with the national and local measures put in place to limit contagion. The instruments we use to tap such stress-related symptoms and the results they produce must thus be interpreted in context. Natural reactions to ongoing stressors must not be interpreted as symptoms of mental disease. What may be surprising to see in this study is that most of the participants seemed to handle the situation (infodemic) well and are only affected to a limited degree.

## Conclusion

Whereas, the experience of the COVID-19 pandemic will pose a stressor to most people, and that as many as 10% in population-based samples may score above cutoff on PTSD screeners, it is unlikely to be a criterion A event for many. However, in the same time we think it is important for clinical practitioners to be aware of the high levels of symptoms reported by some individuals after experiencing COVID-19 lockdown.

## Data Availability Statement

Our data is part of an ongoing longitudinal study and contain potentially sensitive information. The consent of the participants of the Behavior Outlook Norwegian Developmental Study (BONDS), as approved by the Regional Committee for Medical and Health Research Ethics in South-East Norway, does not include sharing a de-identified data set on an open server. Data will therefore be available on request from the Norwegian Center for Child Behavioral Development (NUBU), Oslo, Norway (mail: post@nubu.no) or the corresponding author for researchers who meet the criteria for access to confidential data.

## Ethics Statement

The studies involving human participants were reviewed and approved by The Norwegian Regional Committee for Medical and Health Research Ethics (South East), approval reference: 12552 Barns sosiale utvikling fortsettelse. The participants provided their written informed consent to participate in this study.

## Author Contributions

AN and HJ developed the study design for the BONDS project and were responsible for the acquisition of the data. TI were main responsible for drafting the manuscript, ran the statistical analyses, and prepared the manuscript for publication. All the authors conceived the conceptual model for this specific study, contributed with drafting and commenting to all parts of the manuscript, were involved in the interpretation of the data, have read and approved the final manuscript, and meet the definition of authorship according to the ICMJE's authorship criteria.

## Funding

This project was funded by The Research Council of Norway through the program Research and Innovation in the Educational Sector (FINNUT) (project number 283438, Socioeconomic risk, learning and development from infancy through early adolescence - SLEDE).

## Conflict of Interest

The authors declare that the research was conducted in the absence of any commercial or financial relationships that could be construed as a potential conflict of interest.

## Publisher's Note

All claims expressed in this article are solely those of the authors and do not necessarily represent those of their affiliated organizations, or those of the publisher, the editors and the reviewers. Any product that may be evaluated in this article, or claim that may be made by its manufacturer, is not guaranteed or endorsed by the publisher.
